# Factors Contributing to Stroke Clinic Follow-Up “No Show”: A Quality Improvement Project

**DOI:** 10.7759/cureus.37105

**Published:** 2023-04-04

**Authors:** Carmela V San Luis, Shreyas Gangadhara, Christa O'Hana S Nobleza

**Affiliations:** 1 Neurology, Guthrie Medical Group, Corning, USA; 2 Neurology, University of Mississippi Medical Center, Jackson, USA; 3 Neurology, Baptist Memorial Hospital /University of Tennessee Health Science Center, Memphis, USA

**Keywords:** healthcare seeking practices, stroke patients, rescheduling, outpatient follow up, no show, factors, stroke clinic

## Abstract

Objective

Controlling modifiable risk factors provides a strong impact on secondary stroke prevention. Stroke outpatient follow-up (OPFU) provides a significant role in assuring these goals are met. However, in our institute in 2018, one out of four patients was not seen in the stroke clinic after their stroke. To increase this ratio, we instituted a performance improvement project (PIP) to determine factors that contribute to OPFU and offered rescheduling after their missed appointment.

Methods

The nurse scheduler called patients labeled as “no-show,” asked for reasons for the missed appointment, and offered rescheduling accordingly. Other data were collected retrospectively.

Results

Of the 53 “no show” patients, most were females, single, Black, uninsured, and had a Modified Rankin Scale (MRS) of 0. Of the 30 patients who participated in the phone interview, a most common reason for “no show” was transportation. Fifteen out of 27 patients kept their rescheduled appointment, increasing patients seen in the clinic by 6.7%.

Conclusion

This PIP determined contributing factors on health care seeking practices of our stroke clinic patients allowing necessary improvements in our institute. Rescheduling increased the number of stroke patients seen in the stroke clinic. Our general neurology ambulatory department consequently adopted this process as well.

## Introduction

It is established that controlling modifiable risk factors provides a strong impact on secondary stroke prevention [1]. Identifying the etiology of stroke also impacts the prognosis and management of stroke; [2] and at times, work up for the cause of stroke may continue in the outpatient setting. Thus, stroke outpatient follow-up (OPFU) provides a significant role in assuring that risk factors are controlled, secondary prevention goals are met, and the cause of stroke is determined; however, not all patients are able to adhere to this follow-up. In a study by Ullberg et al., they found that one out of four patients will not be reassessed for adherence to medications and risk factor control [3]. Another study showed 14% of their patients did not see a healthcare professional at all [4]. In our institute, the University of Mississippi Medical Center (UMMC) stroke clinic visit “no-show” rate for 2018 is 27.5%, far from UMMC’s allowable rate of 10%.

Few studies have been done to look at stroke patient factors associated with non-follow-up, and these have shown inconsistent results [3-5]. We believe that understanding the factors that drive healthcare-seeking behaviors in stroke clinics are relevant to make changes in our policies and protocols to improve OPFU compliance. To increase the ratio of patients seen after their stroke, we instituted a performance improvement project (PIP) to determine factors that may contribute to OPFU non-compliance through telephone interviews and retrospective data collection; and offered rescheduling after the missed appointment.

## Materials and methods

Patients and definitions

This PIP was undertaken at the University of Mississippi Medical Center (UMMC) stroke clinic. Because this was a PIP, an Institutional Review Board exemption was granted (IRB file number 2021V0558). All patients who missed their OPFU scheduled in the stroke clinic from January 14, 2019 to February 21, 2019 were labeled as “no shows” and were included.

Quality improvement process

We used the Plan-Do-Study-Act method for improving OPFU in the stroke clinic.

Data collection and analysis

We prospectively determined the most common reason for non-follow-up through a telephone interview. After obtaining verbal consent, the nurse scheduler collected patient information in the provided data collection sheet (Appendix 1). At the end of the interview, patients were asked if they would like to reschedule and were rescheduled accordingly. We then retrospectively obtained the following data: age, sex, marital status, ethnicity, prior no-shows, having a primary care physician (PCP), insurance status, type of insurance, distance, follow-up type, living situation, comorbidities (afib, DM, hypertension, prior stroke), modified Rankin scale (MRS) at discharge, NIH Stroke Scale (NIHSS) at discharge, discharge facility, and intensive care unit (ICU) stay. Descriptive statistics were done utilizing proportions and means as appropriate. We determined the number of patients who kept their appointment after rescheduling and recalculated the increase in patients seen by the neurologist to determine the impact of the intervention. The data obtained were used and discussed with the stroke clinic administrators to assess potential areas of improvement for increasing OPFU compliance. After observing an increase in patients seen, we implemented calling and rescheduling patients in the General Neurology ambulatory clinic as well. We also discussed other factors determined in this study to affect the OPFU rate and utilized this information to create plans for further improvements in the scheduling and follow-up system. The Standards for Quality Improvement Reporting Excellence were utilized in preparing this manuscript.

## Results

A total of 53 patients were labeled as “no shows.” The majority of “no shows” were female, single, Black, had a prior history of “no shows,” or did not have insurance (Table 1). Only four records mentioned the patient's living situation; thus, this category was not included in the analysis. It was also observed that the majority of the patients had two or more comorbidities and most had modified Rankin scale (MRS) of 0, NIHSS of ≤ 8, and no ICU stay or discharged home. Most of the clinic appointments were follow-up visits. It was also noted that scheduling errors contributed to the OPFU rate: six patients did not have stroke diagnoses, one patient had a follow-up scheduled two days from the last follow-up, and three patients had follow-up earlier than what was ordered.

**Table 1:** Demographics

Patient Characteristics		
Age (mean± SD, mode)		53 ± 13 years, 63
Sex (%, n)		
	Female	64.15%, 34
	Male	35.85%, 19
Marital Status (%, n)		
	Single	48.08%, 25
	Married	26.92%, 14
	Widow/ Legally Separated/ Divorce	25%, 13
Race (%, n)		
	Caucasian	26.42%, 14
	Black	69.81%, 37
	Hispanic	3.77%, 2
Current smoker (%, n)		23.91%, 11
Current illicit drug use (%, n)		4.35%, 2
With PCP (%, n)		78.85%, 41
Prior no shows (%, n)		
	No history of no show	80.39%, 41
	With 1 ‘no show’ prior to schedule	13.73%, 7
	With 2 ‘no show’	3.92%, 2
	With 3 ‘no show’	1.96%, 1
Insurance (%, n)		
	None	30.77%, 16
	Medicaid	19.23%, 10
	Medicare	19.23%, 10
	Other insurance (Wellcare, Magnolia, BCBS, United health, etc.)	30.78%, 16
Distance		49.6 ± 45.74 miles
Clinic Visit type		
	Discharge from hospital	21.57%, 11
	New patient	1.96%, 1
	Follow-up from last visit	76.47%, 39

Only 30 patients answered the phone (Table 2) and a common answered reason for non-follow-up is “no transportation” (26.67%, n=8; Table 3). Twenty-seven patients agreed to reschedule and 15 (56%) showed up for the rescheduled date, increasing patients seen by 6.7%. This intervention was applied to the whole Neurology ambulatory clinic after reassessing the PDSA cycle, resulting in increased general neurology OPFU of 8.87%.

**Table 2:** Patient responses

Responses	%, n
Forgot	10, 1
Reschedule	3.33, 1
Not aware of appt	6.67, 2
Wants closer	3.33, 1
Transportation	26.67, 8
Admitted / Accident	10, 3
Too weak, sees PCP	3.33, 1
Seen by other Neurologist	3.33, 1
No insurance	6.67, 2
Feels no need	3.33, 1
Work	6.67, 2
No ID	3.33, 1
Not desired schedule	3.33, 1
Already had follow up	3.33, 1
Bad weather	3.33, 1
Waited for test result	3.33, 1

**Table 3:** Comorbidities and patient disability factors

Comorbidites or disability	%, n
Atrial Fibrillation	4.35%, 2
Hypertension	86.96%, 6
Diabetes mellitus	39.13%, 18
History of stroke	69.57%, 32
Multiple comorbidities (≥ 2)	78.26%, 36
Anticoagulant use	21.74%, 10
Discharge to home	57%, 8
ICU stay	36%, 5
MRS at discharge	
0	38.89 %, 14
1	19.44 %, 7
2	5.56 %, 2
3	16.67 %, 6
4	16.67 %, 6
5	2.78 %, 1
NIHSS at discharge	
Mild (≤8)	91.18 %, 31
Moderate (9–15)	5.88 %, 2
Severe (≥16)	2.94 %, 1

## Discussion

Healthcare utilization, such as going to a scheduled clinic visit, according to Andersen’s Behavioral Model of health services is said to be dependent on 1) predisposing factors (the predisposition of an individual to use services), such as age, sex, marital status, and employment; 2) enabling factors (the ability of the individual to secure services) such as income, insurance, social support, geographic variables and characteristics of the health care system; and 3) need factors, referring to the illness level determined by disease severity, symptom severity, disease duration, comorbidity, and to the perceived need factors, such as overall quality of life, perceived health, activities of daily living and depression [6]. A systematic review of studies that utilized this model to look at healthcare-seeking practices found that women, those older than 39 years old, and were married were more likely to seek medical care [7]. However, our patients' age and gender proportions are inconsistent with this. Looking at the demographics of Jackson, Mississippi, where our institute is located, it was noted that the majority of the inhabitants have an age range of 18-64, who are Black (50%), female (52%), single (54%), with a household income of less than 50,000$ (47%) [8]. This potentially explains the demographic profile of our patients. Other possible predisposing factors that we found in our interview were: “forgot,” “not aware of appointment,” “having work,” “not desired schedule,” and “waited for the test result.” To improve OPFU compliance, we have discussed with the administrators increasing automated schedule reminders to give the patients more opportunity to reschedule ahead of time if their clinic appointment interfered with work or was not their desired time.

Analyses of enabling factors for healthcare-seeking practices found that adults with lower income with less access to alcohol, drug, and mental health care were more likely to not be seen by a doctor [9]. Consistently, the majority of the patients did not smoke or use illicit drugs. However, we believe this is more likely due to patient education of the risks of these stroke recurrences rather than economic factors. Those who had PCPs and regularly followed up with them, in theory, should have a more regular healthcare service use [9,10]. However, 78.85% of our patients did have PCPs yet were still lost to follow-up. Having a PCP listed in the records though does not necessarily indicate they regularly consult them. Also, Redfern et al. found that after patients’ first stroke, 51% saw a specialist; 72% saw a general practitioner, 14% a community nurse, and 14% did not see a doctor at all [4]. Thus, there is a possibility that consulting their PCP may dismiss the necessity to see a Neurologist. Consistently, some patient responses for non-follow-up were “already had follow-up” and “too weak, see PCP.” It is our opinion that if patients are regularly being seen by a healthcare provider and following stroke secondary preventive measures, there may be no need to intervene in these instances.

Regarding insurance, several studies found that being insured increased healthcare services use [11], and subsequently, a majority of our patients lost to follow-up did not have insurance. One of the patients also said their reason for missing the appointment was “no insurance.” Our institute provides financial aid for patients without insurance. However, factors that are involved in the application and approval of these are numerous and beyond our discussion.

Our main hypothesis is that the main enabling factor that contributes to missed appointments is a lack of transportation rather than a source of health funds. Accordingly, most of the patients’ response was “not having transportation.” Other responses that were consistent with this were “wants closer,” “admitted/accident,” and “bad weather.” This data allowed us to determine changes in schedule reminders to better utilize insurance-provided transportations.

Part of the enabling factors is the structure of the hospital and health care system. We have found that there were some errors in scheduling since a couple of patients were scheduled sooner than what was expected, and some did not have a stroke diagnosis. This could have contributed to the OPFU rate. Also, with some patients saying that they have “rescheduled,” “already had to follow up,” “seen by other neurologist,” and “waited for test result,” a possible system that removes prior schedule when a reschedule has been done, requesting a follow-up after a certain test result, or having an alert if another neurologist was seen was proposed. We believe that these factors are important since having an inappropriate “no-show” label could count against the patient’s compliance record. These areas of improvement were also discussed with the administrators. While these other changes are underway, we have been able to implement calling and offering rescheduling after missing an appointment to the General Neurology ambulatory clinic since this intervention increased the number of patients seen by the Neurologist.

Factors that contribute to perceived need such as poorer physical and mental health, activity restriction, and chronic conditions such as hypertension, diabetes mellitus, and high cholesterol were predictors of increased healthcare utilization [9,11,12] Consequently, the majority of our patients were the ones most abled, with MRS of 0, mild NIHSS score, discharged home, and did not have any ICU stay. We hypothesize that this could be due to the lack of a “perceived need” to see a doctor. One patient answered “feels no need” to be seen. We have yet to determine whether a telehealth visit would be more amenable for these patients, especially now that there is increased utilization of telehealth.

It has to be noted that the small sample size and inherent selection bias of only including “no-show” patients limit the generalizability of this project and limit determining associations of the studied factors. Also, the retrospective part of the protocol being dependent on chart reviews is bound by its limitations of documentation. With these in mind, future projects may look further into the factors of OPFU in larger sample sizes and possibly compare these with patients who have kept their appointments with the goal of determining modifiable factors we can improve on.

## Conclusions

This PIP allowed us to assess the predisposing, enabling, and need factors of our stroke clinic patients who missed their OPFU visit. This facilitated understanding of modifiable system-wide processes and factors affecting stroke OPFU non-compliance, determining potential areas of improvement to increase OPFU rate, not just in the stroke clinic, but in our ambulatory neurology department as well. We were also able to improve a number of patients seen in the neurology clinic by calling and offering rescheduling after a missed appointment.

## Figures and Tables

**Figure 1:**
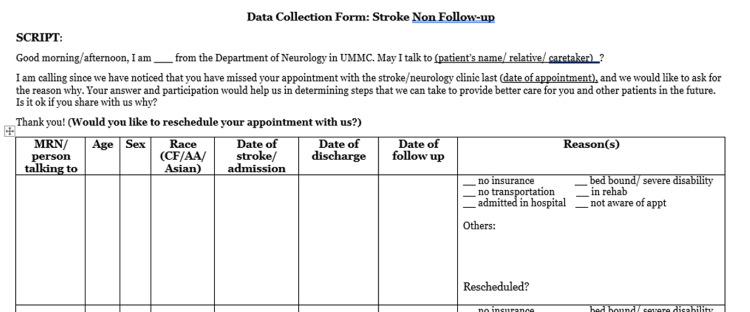
Interview script and data collection form
